# Carbapenem-resistant *Citrobacter freundii* harboring *bla*_KPC−2_ and *bla*_NDM−1_: a study on their transferability and potential dissemination via generating a transferrable hybrid plasmid mediated by IS*6100*

**DOI:** 10.3389/fmicb.2023.1239538

**Published:** 2023-08-17

**Authors:** Feilong Zhang, Ziyao Li, Xinmeng Liu, Yanning Hu, Jiankang Zhao, Yulin Zhang, Yanyan Fan, Zichen Lei, Xinrui Yang, Zhihua Li, Chen Li, Yongli Wu, Binghuai Lu

**Affiliations:** ^1^Peking University China-Japan Friendship School of Clinical Medicine, Beijing, China; ^2^Laboratory of Clinical Microbiology and Infectious Diseases, Department of Pulmonary and Critical Care Medicine, Center of Respiratory Medicine, National Clinical Research Center for Respiratory Diseases, National Center for Respiratory Medicine, China-Japan Friendship Hospital, Beijing, China; ^3^Institute of Respiratory Medicine, Chinese Academy of Medical Sciences, Beijing, China; ^4^China-Japan Friendship Institute of Clinical Medical Sciences, Beijing, China; ^5^Peking Union Medical College, Chinese Academy of Medical Sciences, Beijing, China; ^6^Liuyang Traditional Chinese Medicine Hospital, Changsha, Hunan, China

**Keywords:** carbapenem-resistant *Citrobacter freundii*, KPC-2- and NDM-1-coproducing CRCF, hybrid plasmid, transposition recombination, IS*6100*

## Abstract

**Introduction:**

The increase in clinical *Enterobacteriaceae* with dual carbapenemase has become a serious healthcare concern. It is essential to characterize the transferability and potential dissemination of *bla*_KPC−2_- and *bla*_NDM−1_-coharboring carbapenem-resistant *Citrobacter freundii* (CRCF).

**Methods:**

Four *bla*_KPC−2_- and *bla*_NDM−1_-coharboring CRCF strains were collected from our surveillance of the prevalence of carbapenem-resistant *Enterobacteriaceae*. The isolates were assessed using species identification, antimicrobial susceptibility testing, conjugation assays, whole-genome sequencing, plasmid stability, and fitness costs. Clonality, genome, plasmidome, and phylogeny were analyzed to reveal potential dissemination.

**Results:**

Three ST523 *bla*_KPC−2_- and *bla*_NDM−1_-coharboring CRCF strains, collected from the same hospital within 1 month, exhibited high homology (both identity and coverage >99%), implying clonal dissemination and a small-scale outbreak. Moreover, the *bla*_KPC−2_ and *bla*_NDM−1_ genes were coharbored on an IncR plasmid, probably generated by a *bla*_KPC−2_-harboring plasmid acquiring *bla*_NDM−1_, in these three strains. Importantly, the IncR plasmid may form a transferable hybrid plasmid, mediated by IS*6100* via transposition, with another IncFII plasmid included in the same *C. freundii* strain. Furthermore, the *bla*_KPC−2_ and *bla*_NDM−1_ of the fourth CRCF strain are located on two different non-transferable plasmids lacking complete transfer elements. Additionally, throughout the course of the 10-day continuous passage, the genetic surroundings of *bla*_NDM−1_ in four CRCF strains were gradually excised from their plasmids after the 8th day, whereas they maintained 100% retention for *bla*_KPC−2_. Genome and plasmidome analyses revealed that *bla*_KPC−2_- or *bla*_NDM−1_-harboring *C. freundii* were divergent, and these plasmids have high homology to plasmids of other *Enterobacteriaceae*.

**Conclusion:**

Clonal dissemination of ST523 *bla*_KPC−2_- and *bla*_NDM−1_-coharboring CRCF strains was detected, and we first reported *bla*_KPC−2_ and *bla*_NDM−1_ concomitantly located on one plasmid, which could be transferred with mediation by IS*6100* via transposition. Continued surveillance should urgently be implemented.

## Introduction

*Citrobacter freundii*, a Gram-negative and facultative anaerobic bacillus, is an opportunistic pathogen and causes diverse infections, including those of the urinary tract, respiratory tract, and bloodstream (Liu et al., 2018). Carbapenemase-producing *C. freundii* (CPCF), carrying *bla*_KPC_ encoding a class A serine β-lactamase KPC or *bla*_NDM_ encoding a class B metallo-β-lactamase NDM, has been increasingly reported in recent years and resulted in a small-scale, sporadic outbreak (Hammerum et al., 2016; Bartsch et al., 2017; Jimenez et al., 2017; Bitar et al., 2019; Babiker et al., 2020). Both KPC-2 and NDM-1, the subclasses of KPC and NDM, respectively, show a broad spectrum of hydrolytic activity against penicillins, cephalosporins, and carbapenems. The former can also hydrolyze monobactams and be inhibited by most β-lactamase inhibitors, such as avibactam and relebactam. By comparison, the latter cannot hydrolyze monobactams and is unresponsive to the above β-lactamase inhibitors (Bush, 2013; Bush and Bradford, 2019). Importantly, *bla*_KPC−2_- and *bla*_NDM−1_-coproducing CPCF strains have been recovered from patient specimens and clinical waste in China, leading to limited options for antibacterial treatment (Feng et al., 2015; Wu et al., 2016; Ouyang et al., 2018; Li et al., 2022). Nevertheless, each of those studies only described a single CRCF strain carrying *bla*_KPC−2_ and *bla*_NDM−1_ on two separate plasmids.

Herein, we identified four *bla*_KPC−2_- and *bla*_NDM−1_-coharboring CRCF strains isolated from urine specimens and renal perfusion fluid of four patients in China. We first identified *bla*_KPC−2_ and *bla*_NDM−1_ concomitantly located on one IncR plasmid of three CRCF strains and also compared their genetic environments in conjunction with data available on NCBI, revealing its potential dissemination mechanism.

## Materials and methods

### Identification of 4 *bla*_*KPC*−2_- and *bla*_*NDM*−1_-coharboring CRCF strains and their clinical data

Four *bla*_KPC−2_- and *bla*_NDM−1_-coharboring CRCF strains were collected during our surveillance of the prevalence of carbapenem-resistant *Enterobacteriaceae* isolates. Species identification was determined using matrix-assisted laser desorption ionization–time of flight mass spectrometry (MALDI-TOF MS) (Bruker Daltonik, Bremen, Germany), as described previously (Lu et al., 2021).

### Antimicrobial susceptibility testing

*In vitro* susceptibility tests were performed using N335 susceptibility cards and the Vitek-2 system (bioMérieux, France), including amikacin, minocycline, doxycycline, ceftazidime, cefepime, piperacillin/tazobactam, aztreonam, levofloxacin, ciprofloxacin, and sulfamethoxazole/trimethoprim. The minimal inhibitory concentrations (MICs) of imipenem, meropenem, tigecycline, colistin, ceftazidime–avibactam, and aztreonam–avibactam were determined using the microdilution broth method (bio-KONT, Ltd. China) with *E. coli* ATCC 25922 as the quality control strain, as we described previously (Zhang et al., 2023). The breakpoint of tigecycline was defined by the U.S. Food and Drug Administration (FDA) (Marchaim et al., 2014). The results of other antimicrobial agents were interpreted following the standards of the Clinical Laboratory Standards Institute (CLSI, 2021) (2021). The production of carbapenemases was determined using the modified carbapenem inactivation method (mCIM) and the EDTA-modified carbapenem inactivation method (eCIM), as recommended by the CLSI 2021 (2021).

### Whole-genome sequencing and bioinformatic analysis

As we described previously (Zhang et al., 2023), WGS was performed using the Illumina HiSeq 2500 platform and the nanopore sequencing method on MinION flow cells (for CPCF strains CF2075, CF2084, CF2085, and CF26019 and the transconjugant *E. coli J53*_K-N, respectively). Raw reads were filtered to remove the low-quality sequences and adaptors using skewer (Jiang et al., 2014) and Porechop (https://github.com/rrwick/Porechop), respectively. *De novo* assembly was performed via the SPAdes Genome Assembler v3.13.1 (Prjibelski et al., 2020) and Unicycler (Wick et al., 2017). Gene prediction for CRCF genomes, including four from this study and three retrieved from the NCBI genome database, was performed using Prokka 1.12 (Seemann, 2014). Insertion sequences were identified using the ISfinder database (Siguier et al., 2006). The antimicrobial resistance genes, multilocus sequence types (MLSTs), and plasmid replicon were analyzed via the CGE server (https://cge.food.dtu.dk/services/). Single-nucleotide polymorphisms (SNPs) were determined using Snippy (https://github.com/tseemann/snippy). Homology analysis was performed using BLASTn, FastANI, and SNP. Linear alignments of *bla*_KPC−2_- and *bla*_NDM−1_-bearing structures were generated using genoPlotR and gggenes in R-4.1.2. The transferability of *bla*_KPC−2_ and *bla*_NDM−1_-carrying plasmids in four CRCF strains was evaluated using *oriT*finder (Li et al., 2018).

### Plasmid conjugation assays

Plasmid conjugation experiments were conducted for four CRCF strains, as described previously (Zhang et al., 2023). Azide-resistant *E. coli* J53 and amikacin-resistant *K. pneumoniae* KP54 were used as the recipient strains. In brief, the four CRCF strains and recipient KP54 were adjusted to a McFarland standard of 0.5 and mixed at a ratio of 1:3, and a 0.1-milliliter aliquot of mixture was transferred into LB broth without antibiotics. After an 18-h incubation at 37°C, 200-ml cultures were streaked onto China blue agar (CBA, addition of rosolic acid as the pH indicator) plates containing both amikacin (16 mg/L) and meropenem (1 mg/L) to screen the *bla*_KPC−2−_ or *bla*_NDM−1_-carrying transconjugants. Similarly, KP54_CF2075K-N and recipient *E. coli* J53 were mixed, and transconjugants carrying *bla*_KPC−2_ or *bla*_NDM−1_ were also selected on CBA plates containing both azide (150 mg/L) and meropenem (1 mg/L). The above transconjugants were confirmed by antimicrobial susceptibility testing (AST), polymerase chain reaction (PCR), and pulsed-field gel electrophoresis (PFGE), respectively (Zhang et al., 2023).

### Plasmid stability and fitness cost of CF2075, CF2084, CF2085, CF26019, and the transconjugants

The plasmid stability and fitness cost were assessed as previously described but with slight modifications (Gao et al., 2020). The stability of CF2075, CF2084, CF2085, CF26019, and the transconjugants was evaluated by a passage experiment. In brief, the aforementioned strains were grown in LB broth and transferred at a 24-h interval for 10 consecutive days (approximately 200 generations), at a 1:1000 dilution, into fresh LB broth. The cultures at the 2nd, 4th, 6th, 8th, and 10th days were serially diluted and streaked onto the antibiotic-free LB agar. Approximately 50 colonies were randomly selected to identify the retention of *bla*_KPC−2_ and *bla*_NDM−1_ using PCR. All the above experiments were conducted in triplicate on different days.

Fitness cost was evaluated by growth curves. In brief, CF2085, CF2085ΔNDM (obtained in the above passage assay), CF26019, CF26019ΔNDM, the recipient KP54, and its transconjugants were cultured and shaken at 200 rpm overnight at 37°C in 10 mL LB broth. The overnight cultures were diluted and incubated at 37°C for 25 h to measure the optical density values (OD_600_). The experiment was repeated two times. The growth curves were estimated using Tukey's multiple comparison tests with a one-way analysis of variance (ANOVA).

### Plasmidome analysis of *bla*_*KPC*−2_- or *bla*_*NDM*−1_-carrying plasmids of *C. freundii*

To better unravel the plasmidome of *bla*_KPC−2_- or *bla*_NDM−1_-harboring plasmids in *C. freundii*, we searched the RefSeq database on NCBI and obtained the intact plasmids harboring *bla*_KPC−2_ (42) or *bla*_NDM−1_ (21) in *C. freundii* worldwide as of 20 November 2022. Blasting was performed with BLASTn and illustrated with the R ggplot2 package.

### Phylogenetic analysis

A phylogenetic tree from relevant plasmids was built using PhyML (Guindon et al., 2010), under model GTR running 1,000 bootstrap replicates, from the alignment results generated by mafft (Kuraku et al., 2013). The maximum-likelihood phylogenetic tree of *bla*_KPC−2_- and *bla*_NDM−1_-coharboring CRCF strains in this study with three complete genomes was built using RaxML (Stamatakis, 2014), running 1,000 bootstrap replicates under model GTR-G, from the alignment generated by SNPs and filtered to remove recombination using Gubbins v2.4.1 (Croucher et al., 2015). Visualization was performed using iTOL (https://itol.embl.de).

### Statistical analyses

Data analyses were performed using GraphPad Prism 8.2.1. One-way analysis of variance was used for assessing significant differences, with a *P-value of* < 0.05 being considered statistically significant.

## Results

### Clinical data for 4 CRCF strains

From 11 September 2020 to 12 October 2020, three *bla*_KPC−2_- and *bla*_NDM−1_-coharboring CRCF strains (CF2075, CF2084, and CF2085) were recovered from urine specimens of three inpatients with congenital hydronephrosis post-operation or urinary infection (aged 8 months to 9 years) at the same urological ward of a tertiary hospital in Henan, China (Table 1). The CRCF strain CF26019 was isolated from the renal perfusion fluid of Patient 4 with chronic kidney disease at another tertiary hospital in Beijing, China, after kidney transplantation (Table 1). The four patients all finally recovered from urological diseases.

**Table 1:** Clinical characteristics of patients with *bla*_KPC−2_- and *bla*_NDM−1_-coharboring CRCF strains.

**Patients**	**CF2075**	**CF2084**	**CF2085**	**CF26019**
Age	8 months	9 years	10 months	43 years
Gender	Male	Male	Male	Male
City	Zhengzhou	Zhengzhou	Zhengzhou	Beijing
Ward	Urology	Urology	Urology	Urology
Underlying conditions	Congenital hydronephrosis	Congenital hydronephrosis	Congenital hydronephrosis	Chronic kidney disease
Surgery	Yes	Yes	Yes	Yes
^a^Time span	3 days	2 days	3 days	3 days
Specimen type	Urine	Urine	Urine	Renal perfusion fluid
Infection type	Urinary infection	Urinary infection	Urinary infection	Urinary infection
Temperature (Tmax) (°C)	38.6°C	37°C	36.8°C	36.6°C
Therapeutic antimicrobial usage	Ceftazidime	Cefoperazone–Sulbactam	Cefoperazone–Sulbactam	Cefoperazone–Sulbactam
Length of stay (days)	7	20	11	16
Outcome	Recovered	Recovered	Recovered	Recovered

^a^Time span: Interval between admission and specimen collection.

Multilocus sequence typing (MLST) revealed that all CRCF strains, CF2075, CF2084, and CF2085, belonged to ST523. The strains CF2084 and CF2085 had >99% coverage and identity to CF2075 by BLASTn and FastANI, >99% vice versa. Further analysis revealed that there were only 6 and 18 single-nucleotide polymorphisms (SNPs) between CF2084 and CF2075 and CF2085 and CF2075, respectively. Moreover, Xbal and S1-PFGE also revealed that CF2075, CF2084, and CF2085 had identical bands (Supplementary Figures S1A, B). Overall, PFGE and WGS both indicated that there was a nosocomial outbreak of ST523 CRCF strains through clonal dissemination.

### Phenotypic and genotypic characterization of resistance in *bla*_*KPC*−2_- and *bla*_*NDM*−1_-coharboring CRCF strains

The CRCF strains CF2075, CF2084, CF2085, and CF26019 showed resistance to imipenem, meropenem, ceftazidime, ceftazidime–avibactam, aztreonam, piperacillin/tazobactam, and cefepime but were susceptible to amikacin, tigecycline, colistin, and aztreonam/avibactam for limited treatment options (Table 2). WGS revealed they all carried *bla*_KPC−2_ and *bla*_NDM−1_, in line with resistance phenotypes. Moreover, CF26019 also harbored *aac(3)-IIa* and *aac(6*′*)-Ib-cr*, resulting in resistance to tobramycin.

**Table 2:** Carbapenemase genes located on plasmids and MICs (mg/L) of CF2075, CF2084, CF2085, CF26019, and the transconjugants^a^.

**Strains**	**Species**	**Carbapenemases**	**IMP**	**MEM**	**CAZ**	**CZA**	**ATM**	**ATM/A**	**TZP**	**FEP**	**AMK**	**TIG**	**CST**	**LEV**	**TOB**
CF2075	*C. freundii*	KPC-2, NDM-1	**32**	**64**	**≥64**	**≥256**	**≥64**	0.125	**≥128**	**16**	≤ 2	≤ 0.5	≤ 0.5	1	≤ 1
CF2084	*C. freundii*	KPC-2, NDM-1	**64**	**64**	**≥64**	**≥256**	**≥64**	0.125	**≥128**	**16**	≤ 2	≤ 0.5	≤ 0.5	1	≤ 1
CF2085	*C. freundii*	KPC-2, NDM-1	**64**	**64**	**≥64**	**≥256**	**≥64**	0.125	**≥128**	**≥32**	≤ 2	≤ 0.5	≤ 0.5	1	≤ 1
CF2085ΔNDM	*C. freundii*	KPC-2	**32**	**16**	**16**	≤ 0.25	**≥64**	0.125	**≥128**	8	≤ 2	≤ 0.5	≤ 0.5	1	≤ 1
CF26019	*C. freundii*	KPC-2, NDM-1	**64**	**128**	**≥64**	**≥256**	**16**	0.125	**≥128**	**≥32**	≤ 2	1	≤ 0.5	**≥8**	**≥16**
CF26019ΔNDM	*C. freundii*	KPC-2	**8**	**4**	8	≤ 0.25	**16**	0.125	**≥128**	2	≤ 2	1	≤ 0.5	**≥8**	**≥16**
KP54	*K. pneumoniae*	-	≤ 0.25	≤ 0.25	**32**	≤ 0.125	**≥64**	0.125	**≥128**	**≥32**	**≥64**	2	≤ 0.5	**≥8**	**≥16**
KP54_CF2075K-N	*K. pneumoniae*	KPC-2, NDM-1	**32**	**64**	**≥64**	**≥256**	**≥64**	0.125	**≥128**	**≥32**	**≥64**	2	≤ 0.5	**≥8**	**≥16**
KP54_CF2084K-N	*K. pneumoniae*	KPC-2, NDM-1	**64**	**64**	**≥64**	**≥256**	**≥64**	0.125	**≥128**	**≥32**	**≥64**	2	≤ 0.5	**≥8**	**≥16**
KP54_CF2085K-N	*K. pneumoniae*	KPC-2, NDM-1	**64**	**64**	**≥64**	**≥256**	**≥64**	0.125	**≥128**	**≥32**	**≥64**	2	≤ 0.5	**≥8**	**≥16**
*E. coli* J53	*E. coli*	*-*	≤ 0.25	≤ 0.25	0.125	≤ 0.125	≤ 2	0.125	≤ 4	0.125	≤ 2	≤ 0.5	≤ 0.5	≤ 0.125	≤ 1
*E. coli* J53_K-N	*E. coli*	KPC-2, NDM-1	**32**	**64**	**≥64**	**≥256**	**≥64**	0.125	**≥128**	**16**	≤ 2	≤ 0.5	≤ 0.5	≤ 0.125	≤ 1

^a^IMP, imipenem; MEM, meropenem; CAZ, ceftazidime; CZA, ceftazidime–avibactam; ATM, aztreonam; ATM/A, aztreonam–avibactam; TZP, piperacillin/tazobactam; FEP, cefepime; AMK, amikacin; TIG, tigecycline; CST, colistin; LEV, levofloxacin; TOB, tobramycin. The bold values indicate the MICs mean resistance.

### Genomic analysis of *bla*_*KPC*−2_- and *bla*_*NDM*−1_-coharboring CRCF strains

**Figure 1:**
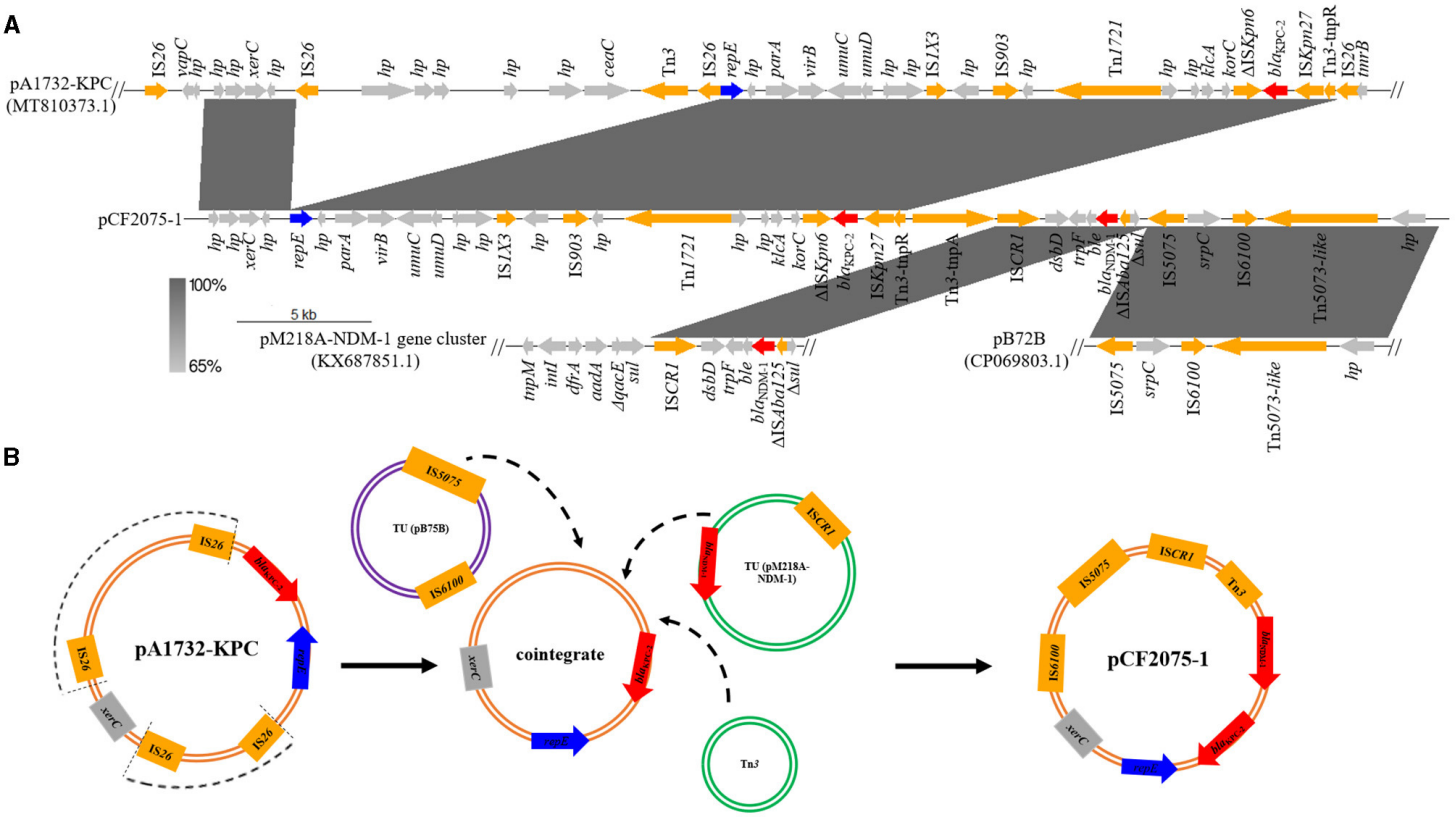
Origin and formation of pCF2075-1. **(A)** Linear sequence alignment analysis on plasmid pA1732-KPC (MT810373.1), pCF2075-1, pM218A-NDM-1 gene cluster (KX687851.1), and pB72B (CP069803.1). **(B)** Proposed formation mechanism of pCF2075-1 as follows. Step 1: pA1732-KPC evolves to generate a cointegrate by losing insignificant segments (arc dotted line) via similar recombination or transposition. Step 2: IS*CR1*, IS*5075*, and Tn*3* mediate the production of transposable elements (TEs), and the cointegrate is integrated into the hybrid plasmid pCF2075-1 by these TEs.

Due to the high homology and clonal spread of the strains of CF2075, CF2084, and CF2085, only CF2075 was chosen for further analysis in detail. WGS and S1-PFGE revealed that CF2075 consisted of a chromosome (5,013,357 bp and 52% GC content) and two plasmids designated as pCF2075-1 (46,049 bp and 56% GC content; identical plasmids pCF2084-1 and pCF2085-1 were identified in CF2084 and CF2085, respectively) and pCF2075-2 (81,274 bp and 53% GC content; identical plasmids pCF2084-2 and pCF2085-2 in CF2084 and CF2085, respectively), classified as IncR and IncFII incompatibility groups, respectively (Supplementary Figures S1A, B). Interestingly, pCF2075-1 concomitantly carried *bla*_KPC−2_ and *bla*_NDM−1_, and the genetic surroundings of the two genes were also contiguous. The *bla*_KPC−2_-harboring genetic environment was Tn*1721*-*klcA*-*korC*-ΔIS*Kpn6*-*bla*_KPC−2_-IS*Kpn27*-Tn*3* (13,760 bp), which is particularly prevalent in China (Figure 1A). It is likely that the *bla*_NDM−1_-harboring genetic context was IS*CR1*-*dsbD*-*trpF*-*ble*-*bla*_NDM−1_-ΔIS*Aba125*-Δ*sul1* (5,250 bp). The *bla*_NDM−1_ gene flanked by IS*CR1* was rarely reported before (Figure 1A). No resistance gene was detected in pCF2075-2.

**Figure 2:**
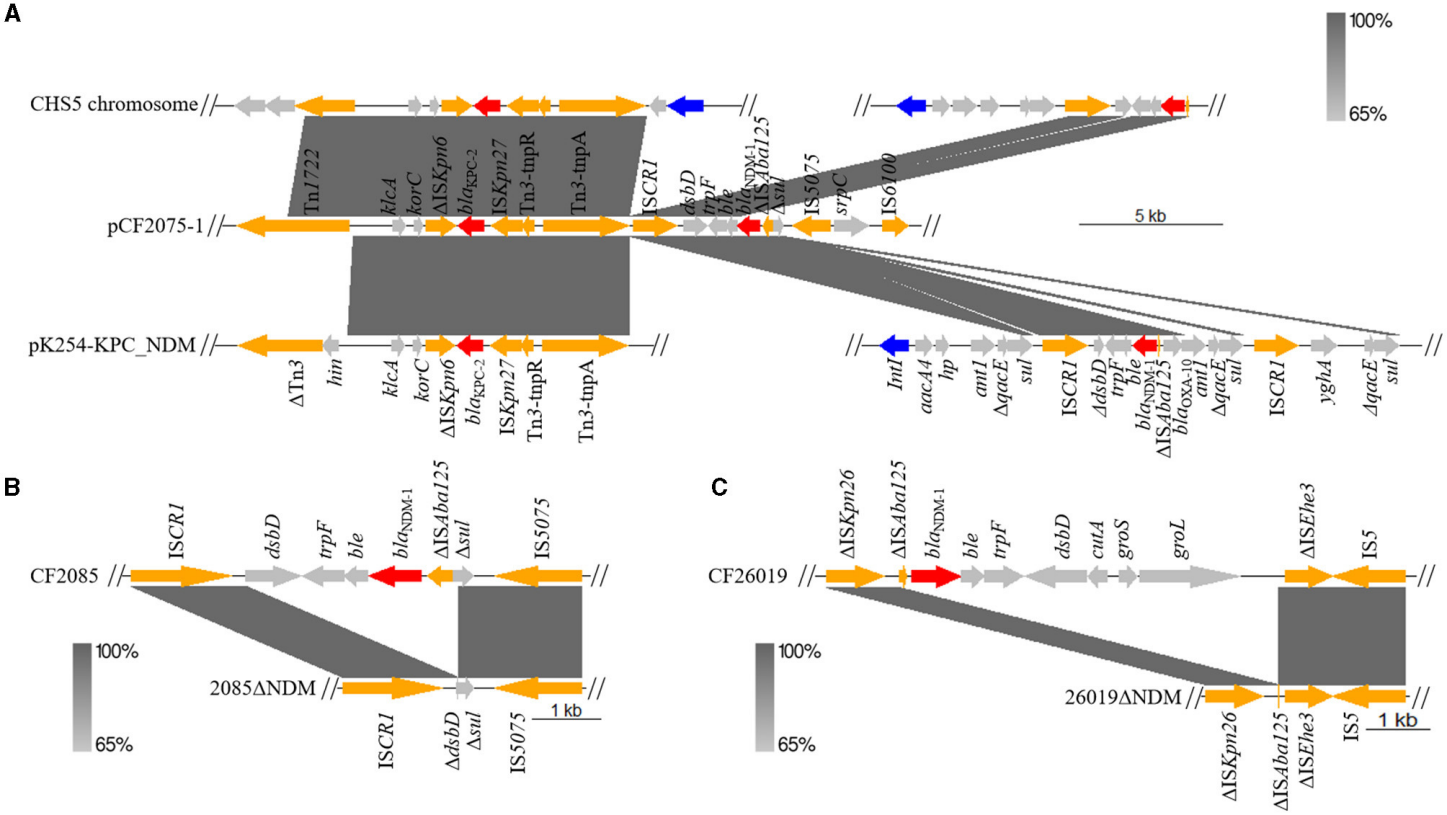
Linear alignment of *bla*_KPC−2_- or *bla*_NDM−1_-bearing plasmid. **(A)** Linear sequence analysis of *bla*_KPC−2_- and *bla*_NDM−1_-bearing chromosome and plasmid on CHS5 chromosome (CP110688.1), pCF2075-1, and pK254-KPC_NDM (OM938013.1). **(B)** Linear comparison of pCF2085-1 between CF2085 and CF2085ΔNDM. CF2085ΔNDM was derived from excision of *bla*_NDM−1_ core structure for CF2085 in conjugation. **(C)** Linear alignment of pCF26019-1 structure on CF26019 and CF26019ΔNDM. CF26019ΔNDM was derived from excision of *bla*_NDM−1_ core structure for CF26019 in conjugation.

To our knowledge, we have first reported the co-existence of *bla*_KPC−2_ and *bla*_NDM−1_ located on one plasmid (pCF2075-1) in *C. freundii*. The plasmid pCF2075-1, an IncR plasmid, exhibited low coverage (< 82.0%) compared to these plasmids on NCBI. Therefore, pCF2075-1 is a previously unreported fusion plasmid. To decipher the forming mechanism of pCF2075-1, its structure was analyzed in detail. It consisted of pA1732-KPC (63.5%, MT810373.1), the pM218A-NDM-1 gene cluster (11.4%, KX687851.1), and pB72B (22.4%, CP069803.1). The pA1732-KPC was contained in *K. pneumoniae* A1732 and harbored the same replicon IncR and *bla*_KPC−2_-harboring genetic context as pCF2075. Consequently, pA1732-KPC could be considered the backbone of pCF2075-1. However, the genetic environment of *bla*_KPC−2_ of pCF2075-1 did not harbor IRL-2 (Tang et al., 2022), which could embed *bla*_KPC−2_ into transposon Tn*1721* for transfer. This transposon is non-transferable by carrying *bla*_KPC−2_. The pM218A-NDM-1 in *Escherichia coli* harbored *bla*_NDM−1_, flanked by IS*CR1* of a class 1 Integron. IS*CR1* can generate a transposable element (TE) mediating the transfer of *bla*_NDM−1_. It implied that the *bla*_NDM−1_-harboring TE was integrated into the backbone of pCF2075-1 to generate the *bla*_KPC−2−_ and *bla*_NDM−1_-co-carrying cointegrate (Figure 1B). Furthermore, the other two convergences of *bla*_KPC−2_ and *bla*_NDM−1_ in the *K. pneumoniae* strain CHS5 chromosome (CP110688.1) and the *K. michiganensis* strain K254 plasmid pK254-KPC_NDM (OM938013.1) were retrieved from NCBI (Figure 2A). The *bla*_KPC−2_-harboring genetic contexts of the CHS5 chromosome and pK254-KPC_NDM were similar to Tn*6296*, which originated from the Tn*1722*-based transposon inserted by the core *bla*_KPC−2_ region. Notably, the *bla*_NDM−1_-harboring genetic contexts of both the CHS5 chromosome and pK254-KPC_NDM were contained in a class 1 Integron, in which the core *bla*_NDM−1_ region was flanked by IS*CR1*. Collectively, a propensity for the co-existence of *bla*_KPC−2_ and *bla*_NDM−1_ was overwhelmingly dominated by the IS*CR1*-mediating transfer of *bla*_NDM−1_ and the Tn*1721*/Tn*6296*-based-transposon-mediating transfer of *bla*_KPC−2_.

In addition, CF26019 belonged to ST118 and contained a chromosome (5,177,371bp and 52% GC content) and four plasmids. Unlike CF2075, *bla*_NDM−1_ and *bla*_KPC−2_ of CF26019 were separately located on plasmid pCF26019-1 (44,169 bp and 56% GC content) and pCF26019-3 (35,420 bp and 57% GC content), belonging to IncFII/IncFIB and IncP6 incompatibility groups, respectively. Furthermore, pCF26019-1 showed a maximum query coverage of 73% with a nucleotide identity of 100% to the plasmids p205880-NDM of *K. pneumoniae* MH909345.1 and pK218-NDM of *C. portucalensis* OL988824.1. The genetic surrounding of *bla*_NDM−1_ on pCF26019-1 was IS*5*-ΔIS*Ehe3*-*groL*-*groS*-*cutA*-*dsbD*-*trpF*-*ble*-*bla*_NDM−1_-ΔIS*Aba125*(8,208 bp), which is a common genetic surrounding observed in *bla*_NDM−1_-harboring *Enterobacteriaceae* strains. The pCF26019-3 exhibited 91% coverage and 100% identity to eight plasmids on NCBI, including CF121SC21 plasmid1 (LT992437.1) of *C. freundii* and plasmid pA1705-KPC (MH909348.1) of *K. pneumoniae* A1705. The genetic context of *bla*_KPC−2_ on pCF26019-3 was ΔIS*Ec33*-Tn*3*-IS*Apu1*-*hp*-IS*Apu2*-IS*Kpn27*-Δ*bla*_TEM − 1_-*bla*_KPC−2_-ΔIS*Kpn6*-*korC-klcA* (13,066 bp), which commonly exists on different plasmids in *Enterobacteriaceae* (MN539620.1, MN477223.1, and MH909348.1) and *Pseudomonas* species (CP040685.1).

### Transferability, stability, and fitness cost of *bla*_*KPC*−2_- and *bla*_*NDM*−1_-coharboring CRCF strains

The transferability of *bla*_KPC−2_ and *bla*_NDM−1_ was evaluated by conjugation assays. The results revealed that *bla*_KPC−2_ and *bla*_NDM−1_ of CF2075, CF2084, and CF2085 could be concomitantly transferred to the recipient *K. pneumoniae* KP54, a clinical strain isolated from urine samples, and the transconjugants were designated as KP54_CF2075K-N, KP54_CF2084K-N, and KP54_CF2085K-N, respectively (Supplementary Figure S1B). Further conjugation experiments revealed that *bla*_KPC−2_ and *bla*_NDM−1_ of KP54_CF2075K-N could also be simultaneously conjugated into recipient *E. coli* J53, designated as *E. coli* J53_K-N (Supplementary Figure S1C). The above transconjugants were verified by resistance phenotype using PCR and PFGE. However, no transconjugant was obtained after co-culturing CF26019 with KP54, even though we repeated the conjugation assay dozens of times.

**Figure 3:**
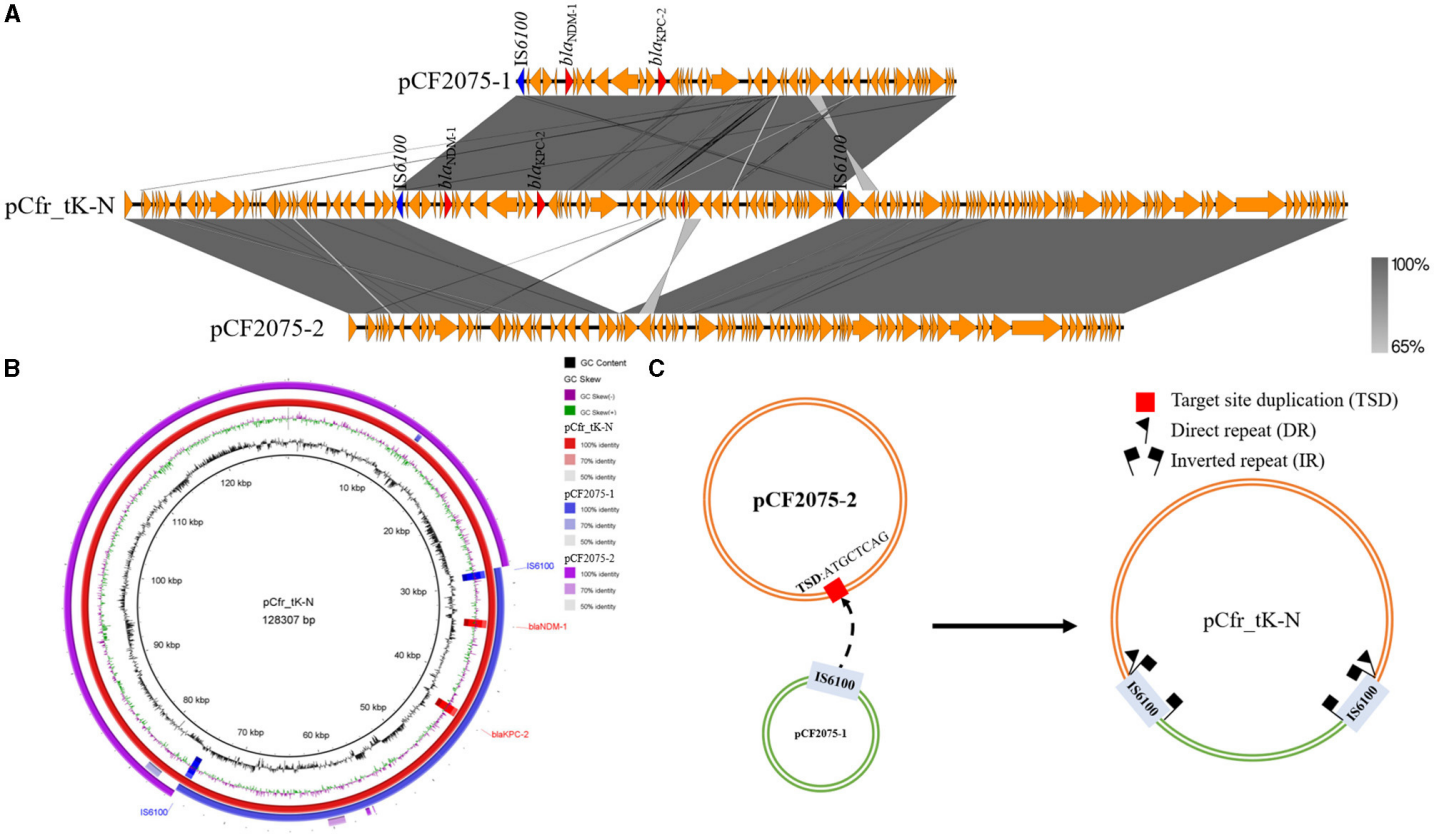
Emergence and recombination mechanism of hybrid plasmid pCfr_tK-N generated during conjugation. **(A)** Linear sequence comparison of hybrid plasmid pCfr_tK-N with pCF2075-1 and pCF2075-2. **(B)** Circular alignment analysis of pCF2075-1, pCF2075-2, and hybrid plasmid pCfr_tK-N. Alignments were generated using the BLAST Ring Image Generator (BRIG). **(C)** Proposed model for the formation of the fusion plasmid pCfr_tK-N mediated by IS*6100* transposition.

Unexpectedly, S1-PFGE showed that all the transconjugants, KP54_CF2075K-N, KP54_CF2084K-N, and KP54_CF2085K-N, contained a novel fusion plasmid co-carrying *bla*_KPC−2_ and *bla*_NDM−1_, named pCfr_tK-N, which is different from any plasmids of CF2075, CF2084, and CF2085 in size. However, the size of the pCfr_tK-N is almost equal to the sum of the size of pCF2075-1 and pCF2075-2; that is to say, pCfr_tK-N was probably generated by the recombination of pCF2075-1 and pCF2075-2. To unravel the forming mechanism of pCfr_tK-N, WGS was performed. Sequence analysis revealed that pCfr_tK-N was indeed the recombination result of pCF2075-1 and pCF2075-2 (Figures 3A, B), and the junctions of these two plasmids were two insertion sequence IS*6100*s. Of note, two 8-base-pair direct repeat (DR, ATGCTCAG) sequences were adjacent to the left inverted repeat sequence (IRL) of one IS*6100* and the right inverted repeat sequence (IRR) of another IS*6100*, which implied that the recombination was mediated by IS*6100* (Figure 3C). The plasmid stability of four CRCF strains and transconjugants was evaluated by passage experiment (Supplementary Figure S1E). Excisions of *bla*_NDM−1_ strains were obtained from CF2085 and CF26019 on the 8th day, named CF2085ΔNDM and CF26019ΔNDM, respectively. However, S1-PFGE and the sequence analysis showed that *bla*_NDM−1_-harboring genetic surroundings were excised from the plasmids of CF2085 and CF26019, which were inconsistent with the loss of *bla*_NDM−1_ usually caused by the removal of plasmids harboring *bla*_NDM−1_ in most cases (Figure 2B and Supplementary Figure S1B). A similar phenomenon was also observed in CF2075 and CF2084 on the 10th day of the passage experiment. However, the transconjugants (KP54_CF2075K-N, KP54_CF2084K-N, KP54_CF2085K-N, and *E. coli* J53_K-N) maintained 100% retention during the process of 10-day continuous passage, showing that the hybrid plasmids harboring *bla*_KPC−2_ and *bla*_NDM−1_ could be stably inherited. In summary, *bla*_KPC−2_- and *bla*_NDM−1_-coharboring CRCF strains and transconjugants can retain the stable inheritance of *bla*_KPC−2_- and *bla*_NDM−1_-co-carrying plasmids (>90% retention in the 10-day passage) that might slow the clearance of resistance genes and facilitate clonal dissemination.

Growth rates were measured to assess the impact of the acquisition of *bla*_KPC−2_- and *bla*_NDM−1_-coharboring plasmids on biological fitness cost. A significant growth difference (*P* < 0.0001) was shown between CF2085 and CF2085ΔNDM, KP54 and KP54_CF2075K-N, and *E. coli* J53 and *E. coli* J53_K-N, whereas no significant difference (*P* > 0.5) was observed in the growth rates between CF26019 and CF26019ΔNDM. Taken together, the *bla*_KPC−2_- and *bla*_NDM−1_-coharboring plasmids exhibited punishment of fitness in CF2075, CF2084, CF2085, and their transconjugants.

### Plasmidome analysis of *bla*_*KPC*−2_-harboring plasmids in *C. freundii*

**Figure 4:**
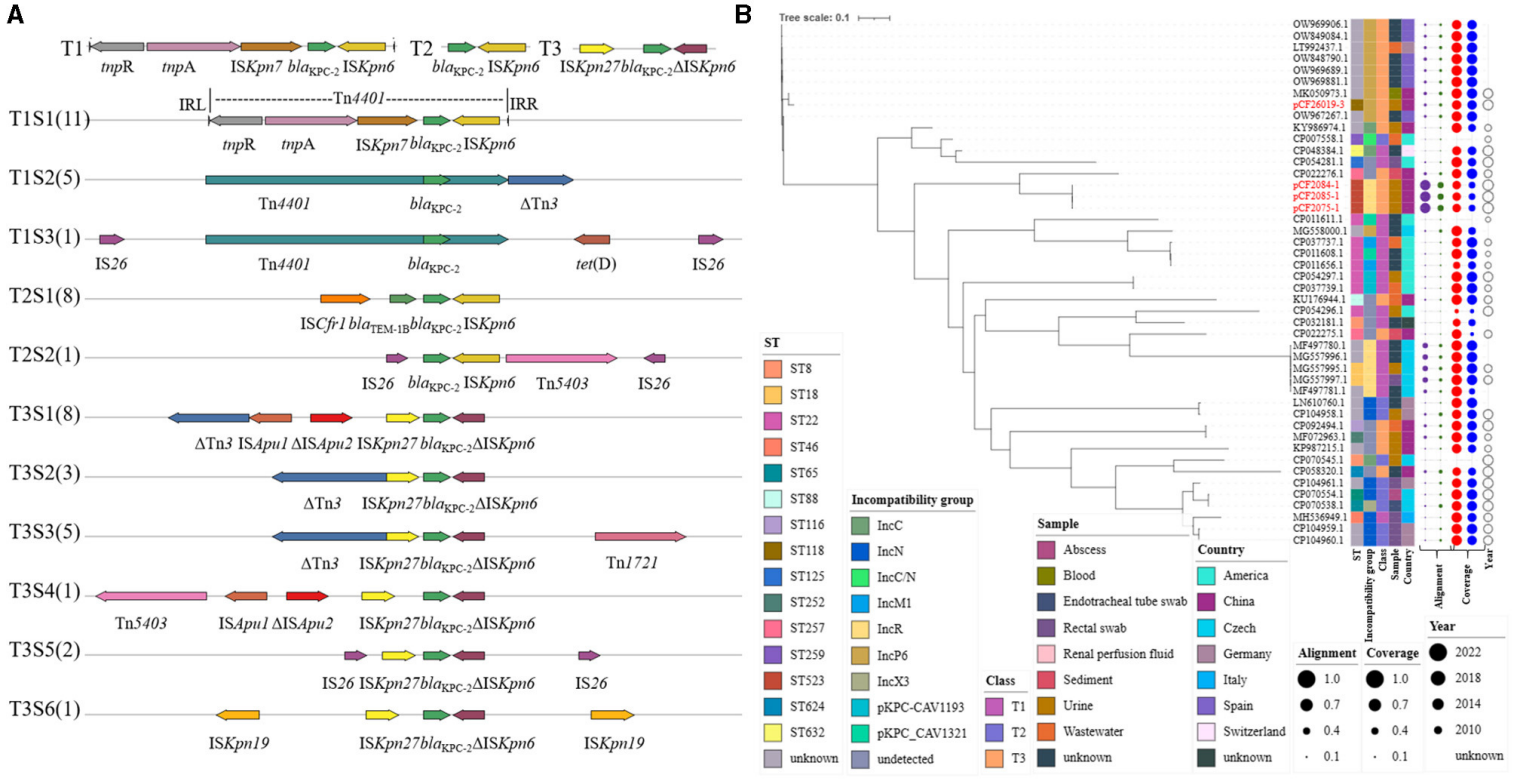
Plasmidome analysis of *bla*_KPC−2_-bearing plasmid in *C. freundii*. **(A)** *bla*_KPC−2_ genetic surrounding was analyzed in *bla*_KPC−2_-bearing plasmid of *C. freundii* strains, including 42 strains from NCBI and 4 from this study. **(B)** The maximum-likelihood phylogenetic tree was built by PhyML from 46 complete plasmids' sequence alignment generated by mafft. The tree was visualized and annotated using Interactive Tree Of Life (iTOL, https://itol.embl.de). Class—different types of *bla*_KPC−2_ genetic surrounding on the left. Alignment—alignment to pCF2075-1 and pK254-KPC_NDM. Coverage—coverage to plasmids in non-*Citrobacter* sp., *Enterobacteriaceae*, and *Citrobacter* sp.

A total of 46 *bla*_KPC−2_-harboring plasmids contained in *C. freundii* strains, including 42 retrieved from NCBI and 4 in this study, were analyzed via phylogenetic tree (Figure 4). These 42 *C. freundii* strains were recovered from urine, sediment, rectal swab, and blood and also from wastewater in the hospital environment and were gathered in Spain, the Czech Republic, China, and USA. Heterogeneous STs were identified in *bla*_KPC−2_-harboring *C. freundii*, including ST8, ST18, ST22, ST65, ST118, ST257, ST259, ST523, and ST632. The *C. freundii* strains belonging to the same STs might carry both closely related plasmids (such as pCF2075-1, pCF2084-1, and pCF2085-1 or CP037739.1 and CP054297.1), largely implying a clonal spread, and distantly related ones (CP011608.1 and CP011656.1). Plasmid incompatibility groups revealed that the above closely related plasmids had the same replicon, including IncP6, IncR, and IncN. Furthermore, *bla*_KPC−2_-harboring plasmids showed ≤ 52% and ≤ 59% coverage compared with the *bla*_KPC−2_- and *bla*_NDM−1_-co-carrying plasmids pCF2075-1 and pK254-KPC_NDM, respectively. Notably, most *bla*_KPC−2_-harboring plasmids (43/46, 93.5%) exhibited ≥82.0% coverage at 100% identity to the plasmids contained in non-*C. freundii Enterobacteriaceae*. Similarly, *bla*_KPC−2_-harboring plasmids (34/46, 74.0%) also exhibited ≥82.0% coverage to the plasmids of *C. freundii*.

### Plasmidome analysis of *bla*_*NDM*−1_-harboring plasmids in *C. freundii*

**Figure 5:**
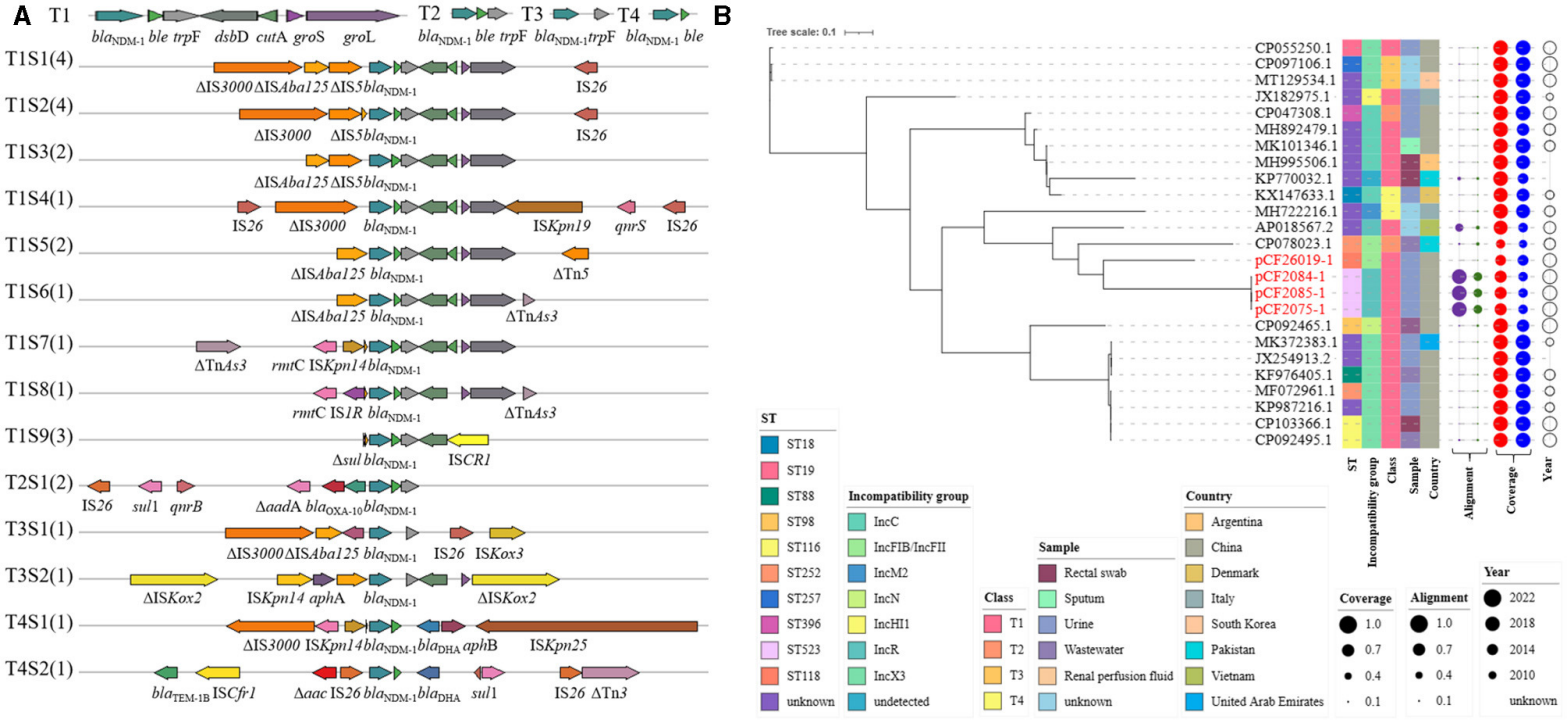
Plasmidome analysis of *bla*_NDM−1_-bearing plasmid in *C. freundii*. **(A)** *bla*_NDM−1_ genetic surrounding was analyzed in *bla*_NDM−1_-bearing plasmid of *C. freundii* strains, including 21 strains from NCBI and 4 from this study. **(B)** The maximum-likelihood phylogenetic tree was built by PhyML from 25 complete plasmids' sequence alignment generated by mafft. The Interactive Tree Of Life (https://itol.embl.de) was used for visualization. Class—different types of *bla*_NDM−1_ genetic surrounding on the left. Alignment—alignment to pCF2075-1 and pK254-KPC_NDM. Coverage—coverage to plasmids in non-*Citrobacter* sp., *Enterobacteriaceae*, and *Citrobacter* sp.

Four *bla*_NDM−1_-harboring plasmids in this study and twenty-one on NCBI were comprehensively analyzed (Figure 5). Their STs also showed diversity, including ST18, ST19, ST88, ST98, ST116, ST252, ST257, ST396, and ST523. Incompatibility groups mainly focus on IncX3 (10/25, 40.0%), IncC (5/25, 20.0%), and IncR (4/25, 16.0%), while identical replicons showed an obvious cluster (MK101346.1 and MH995506.1; CP097106.1 and CP055250.1; and pCF2075-1, p-CF2084-1, and pCF2085-1). However, a similar core *bla*_NDM−1_ region was not always contained in a cluster. The *bla*_NDM−1_-harboring plasmids were isolated from clinical species and environments in line with *bla*_KPC−2_-harboring plasmids and were mainly from China (16/25, 64.0%) since 2010. In contrast with pCF2075-1 and pK254-KPC_NDM, all *bla*_NDM−1_-harboring plasmids exhibited ≤ 55% coverage. To better evaluate the origin of *bla*_NDM−1_-harboring plasmids in *C. freundii*, these plasmids were compared with those on NCBI. The results revealed that 72.0% (18/25) plasmids in *C. freundii* and 92.0% (23/25) plasmids in other *Enterobacteriaceae* shared >80% of their length with the aforementioned *bla*_NDM−1_-containing plasmids, respectively.

### Genome analysis of 7 *bla*_*KPC*−2_- and *bla*_*NDM*−1_-coharboring CRCF strains

**Figure 6:**
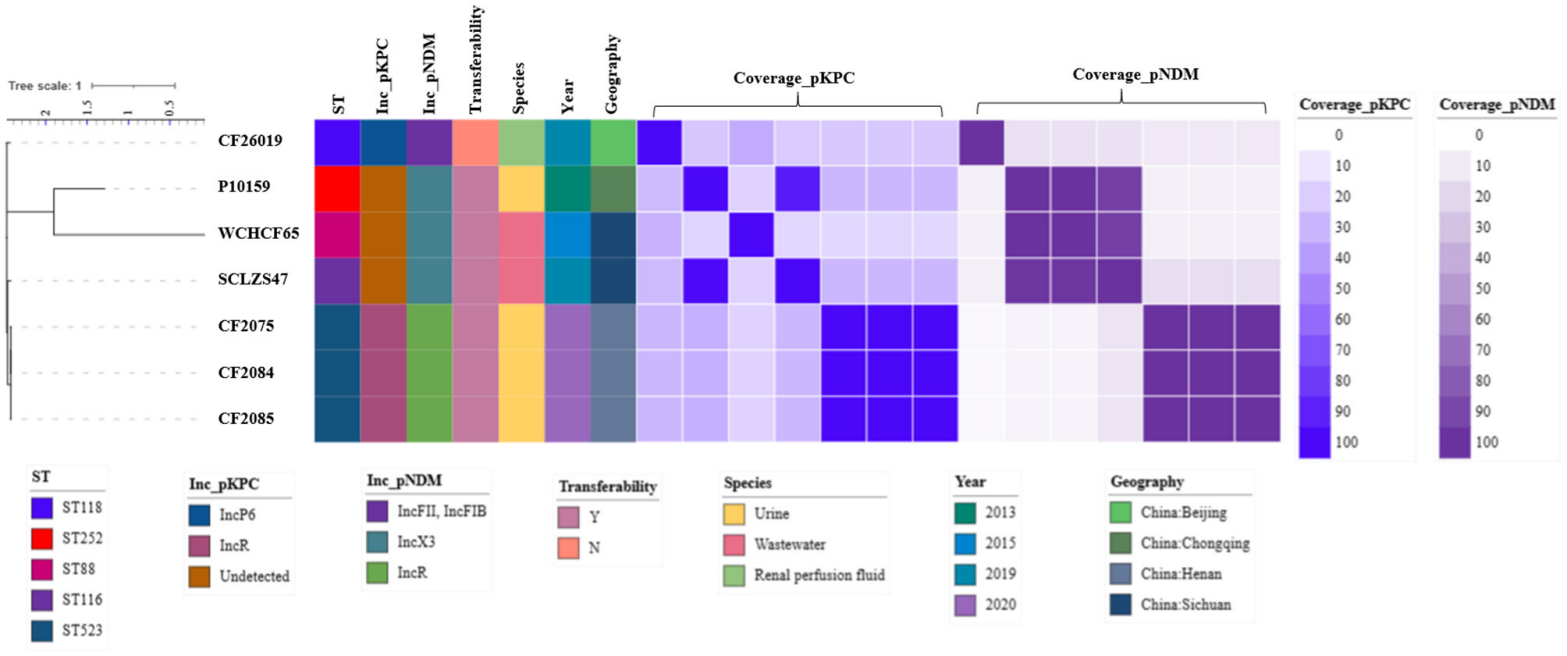
Maximum-likelihood phylogenetic tree of seven *bla*_KPC−2_- and *bla*_NDM−1_-coharboring CRCF strains was built by RaxML. The tree is annotated based on ST, Inc_pKPC (incompatibility group of *bla*_KPC−2_-bearing plasmids), Inc_pNDM (incompatibility group of *bla*_NDM−1_-bearing plasmids), transferability, species, year, geography, and similarity among *bla*_KPC−2_-bearing plasmids (blue heatmap) and *bla*_NDM−1_-bearing plasmids (purple heatmap) in *C. freundii*. The similarity is defined as the coverage of homology regions for query plasmid (row-wise) and subject plasmid (column-wise).

To decipher the underlying mechanism in forming *bla*_KPC−2_- and *bla*_NDM−1_-coharboring CRCF strains, seven *bla*_KPC−2_- and *bla*_NDM−1_-coharboring CRCF strains having complete genomes, including three reported previously (Wu et al., 2016; Ouyang et al., 2018; Li et al., 2022) and four in this study, were analyzed (Figure 6). All were recovered in China after 2013 and could be divided into three clusters based on genome (cluster 1: CF26019; cluster 2: P10159 and WCHCF65; cluster 3: CF2075, CF2084, CF2085, and SCLZS47). STs showed an extensive distribution, including ST88, ST116, ST118, ST252, and ST523.

The *bla*_KPC−2_-harboring plasmids in both P10159 and SCLZS47 were identical, although not grouped into the same cluster, and CF2075, CF2084, and CF2085 also contained the same plasmids. Therefore, four different *bla*_KPC−2_-harboring plasmids were identified in the seven CRCF strains. These different plasmids, other than the type of pCF2075-1, have a high coverage of similar sequence (bi-directional ≥90%) in *Enterobacteriaceae*. For *bla*_NDM−1_-harboring plasmids, three different plasmid types were identified. It is likely that the coverage of similar sequences among these plasmids was also distantly related (4.0–16.0%). Only the type of pP10159-1 (MF072961.1) had similar plasmids (bi-directional coverage and identity ≥99.9%) reported before in *Enterobacteriaceae*, and others were novel ones. Furthermore, P10159 and SCLZS47 were isolated from Chongqing and Sichuan in China, which are geographically close, and had identical *bla*_KPC−2_- and *bla*_NDM−1_-harboring plasmids, but the chromosomes of P10159 had 81.0% coverage of similar sequence at 95.94% to SCLZS47 and 80.0% the other way around, hinting at a horizontal gene transfer (HGT) by plasmids.

## Discussion

CRCF coharboring *bla*_KPC−2_ and *bla*_NDM−1_ was first reported in 2015 (Feng et al., 2015) and has emerged continually in recent years, especially in China (Feng et al., 2015; Wu et al., 2016; Ouyang et al., 2018; Li et al., 2022). However, comprehensive analyses for the CRCF strains were lacking, and the potential dissemination mechanism has remained unclear.

In the current study, four *bla*_KPC−2_- and *bla*_NDM−1_-coharboring CRCF strains were identified. Three of them (CF2075, CF2084, and CF2085), recovered from urine samples within 1 month in the same ward, indicated a clonal outbreak according to PFGE, WGS, and phylogenetic tree analyses. WGS revealed a *bla*_KPC−2_- and *bla*_NDM−1_-coharboring plasmid in these three CRCF strains, which was first reported here. The clonal outbreak of coharboring dual carbapenemase genes will pose a severe threat to public health. Only the plasmid pK254-KPC_NDM (OM938013.1) in *K. michiganensis* and the chromosome (CP110688.1) CHS5 in *K. pneumoniae* are coharboring *bla*_KPC−2_ and *bla*_NDM−1_, in line with the data on NCBI. The *bla*_NDM−1_-carrying genetic surroundings of the above plasmid and chromosome were highly identical, facilitating the generation of TE and further spread. However, the *bla*_KPC−2_-carrying genetic surroundings of them were Tn1721/Tn6296-based transposons, which were usually generated in an ancestor transposon by inserting another transposon, and lacked an intact inverted repeat sequence (IR) at both sides of the core *bla*_KPC−2_ region, resulting in difficult transfer. Taken together, it is reasonable to hypothesize that a *bla*_KPC−2_- and *bla*_NDM−1_-coharboring plasmid or chromosome might be derived from *bla*_KPC−2_-harboring plasmid or chromosome progenitors that acquired *bla*_NDM−1_ by HGT, such as TUs and transposons. AST revealed that aztreonam–avibactam was probably an option for *bla*_KPC−2_- and *bla*_NDM−1_-coharboring CRCF strains.

To evaluate the transferability of *bla*_KPC−2_ and *bla*_NDM−1_, a series of transconjugants were obtained by conjugation assays and showed consistency of resistance phenotype and genotype. However, S1-PFGE showed that pCF2075-1 was not transferred alone to the recipient, and further analysis revealed that during conjugation, the fusion plasmid pCfr_tK-N was generated and could be transferred to the recipient strains KP54 and *E. coli* J53, respectively. Sequence analysis indicated that pCfr_tK-N was a recombinant of pCF2075-1 and pCF2075-2 mediated by IS*6100*, and this intermolecular transposition was first reported. Similar recombination, mediated by IS*26*, IS*Kpn14*, IS*Kpn74*, and IS*903B*, was also reported in conjugations among different resistant or virulent plasmids in recent years, which could accelerate the dissemination of resistance and virulence genes (Wang et al., 2022a,b; Yang et al., 2022). The pCF2075-1 being transferred by generating recombinants might be explained by the fact that the self-transferrable plasmids usually contain four modules: origin of transfer site *oriT*, relaxase gene, gene encoding type IV coupling protein (T4CP), and gene cluster for bacterial type IV secretion system (T4SS). Putative transferability analysis revealed that pCF2075-1 only contained *oriT* and relaxase gene, which prevented it from being transferred alone. However, pCF2075-2 contained all four complete modules. Therefore, pCF2075-1 is required to be integrated into pCF2075-2 to be transferrable. Furthermore, the stability and fitness cost of the CRCF strains and transconjugants were evaluated. These exhibited higher retention for *bla*_KPC−2_ and *bla*_NDM−1_ (>90.0% after 10 days) but required biological cost.

Plasmidome is extremely essential for understanding its origin and taking measures to prevent its propagation. In total, 46 *bla*_KPC−2_-harboring plasmids and 25 *bla*_NDM−1_-harboring plasmids retrieved from NCBI were systematically analyzed. Analysis revealed that *C. freundii* strains with *bla*_KPC−2_- or *bla*_NDM−1_-harboring plasmids shared the same STs, such as ST18 and ST257, that might generate more *bla*_KPC−2_- and *bla*_NDM−1_-coharboring CRCF strains in future. Importantly, most *bla*_KPC−2_- or *bla*_NDM−1_-harboring plasmids in *C. freundii* had high homology to the plasmids of other *Enterobacteriaceae*, hinting at the high transferability of *bla*_KPC−2_- or *bla*_NDM−1_-harboring plasmids. Moreover, the types of core *bla*_KPC−2_ and *bla*_NDM−1_ regions were relatively conserved, although *bla*_KPC−2_ or *bla*_NDM−1_ genetic surroundings exhibited multiple types by different ISs, indicating that ISs play a significant role in driving resistance genes' transfer.

We next comprehensively elaborated on the evolutionary relationships of seven *bla*_KPC−2_- and *bla*_NDM−1_-coharboring CRCF strains. These were grouped into three clusters. Notably, P10159 and SCLZS47 had the same *bla*_KPC−2_- and *bla*_NDM−1_-harboring plasmids, although they did not belong to the same cluster. The discordance of homology of *bla*_KPC−2_- or *bla*_NDM−1_-harboring plasmids to the genome evolution indicated that these have a strong ability to transfer and adapt to different hosts. As mentioned above, *bla*_KPC−2_- or *bla*_NDM−1_-harboring plasmids in *C. freundii* belong to diverse STs. Therefore, the formation of *bla*_KPC−2_- and *bla*_NDM−1_-coharboring CRCF strains probably occurs in two ways: *bla*_KPC−2_-harboring *C. freundii* acquires *bla*_NDM−1_-harboring plasmids, or *bla*_NDM−1_-harboring *C. freundii* acquires *bla*_KPC−2_-harboring plasmids.

In conclusion, we identified four *bla*_KPC−2_- and *bla*_NDM−1_-coharboring CRCF strains and reported, for the first time, *bla*_KPC−2_ and *bla*_NDM−1_ concomitantly located on one plasmid. Notably, the plasmid was integrated into another plasmid to generate an uncommon fusion plasmid, mediated by IS*6100* via transposition, which could be transferred into a different genus in *Enterobacteriaceae*. Genome and plasmidome analyses revealed that *bla*_KPC−2_- or *bla*_NDM−1_-harboring *C. freundii* were divergent, and these plasmids have high homology to plasmids of other *Enterobacteriaceae*.

## Data availability statement

The datasets presented in this study can be found in online repositories. The names of the repository/repositories and accession number(s) can be found below: https://www.ncbi.nlm.nih.gov/, BioProject: PRJNA937448.

## Author contributions

FZ and ZiyL performed the experiments. FZ, ZiyL, and XL analyzed the data. FZ and BL wrote the main manuscript text and prepared Figures 1–6 and Supplementary Figure S1. ZiyL, XL, YH, YW, JZ, YZ, YF, ZicL, XY, ZhiL, and CL prepared Tables 1, 2. All authors reviewed the manuscript. All authors contributed to the article and approved the submitted version.

## Supplementary material

Supplementary Figure S1 PFGE, the stability and growth curves of CF2075, CF2084, CF2085, CF26019, and the transconjugants. **(A)** Xbal-PFGE of CF2075, CF2084, CF2085, CF26019, and the transconjugants. **(B, C)** S1-PFGE of these strains. Red arrows indicate transferable hybrid plasmid pCfr_tK-N. **(D)** PCR for *bla*_KPC−2_ and *bla*_NDM−1_ and excision sequences of CF2085, CF2085ΔNDM, CF26019, and CF26019ΔNDM. Green arrows indicate the remains of pCF2085-1 and pCF26019-1 excising of *bla*_NDM−1_-bearing surrounding in CF2085ΔNDM and CF26019ΔNDM, respectively. **(E)** The stability of these strains throughout the course of the 10-day continuous passage. **(F)** Growth curves of these strains within 25 h.
