# Supplementary figures and images for: Carbapenem-resistant Citrobacter freundii harboring blaKPC−2 and blaNDM−1: a study on their transferability and potential dissemination via generating a transferrable hybrid plasmid mediated by IS6100
Source: Front Microbiol. 2023 Aug 17;14:1239538. doi: 10.3389/fmicb.2023.1239538 (PMC10469622; doi:10.3389/fmicb.2023.1239538)

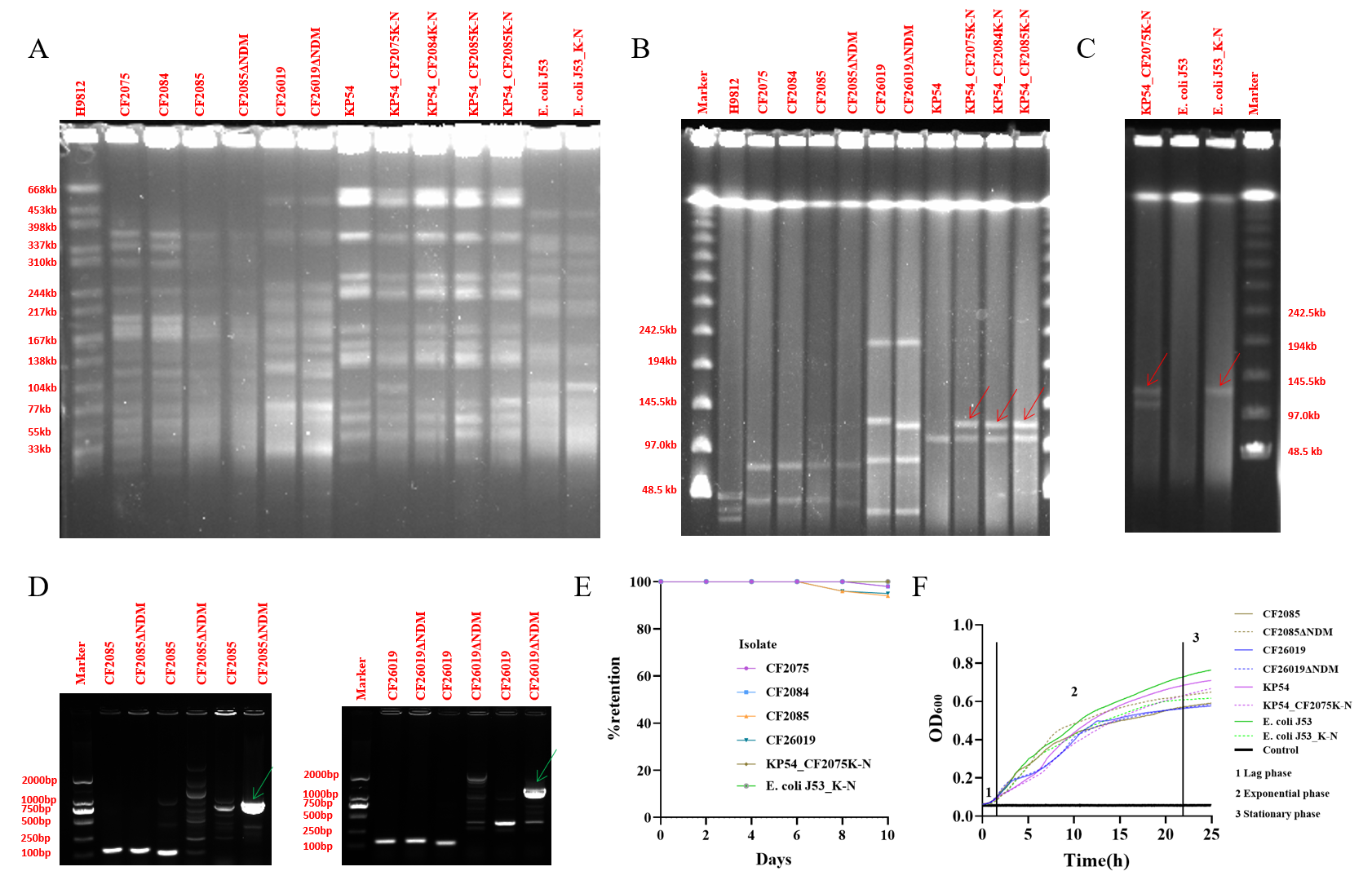

Supplement: Supplementary Figure S1 — PFGE, the stability and growth curves of CF2075, CF2084, CF2085, CF26019, and the transconjugants. (A) Xbal-PFGE of CF2075, CF2084, CF2085, CF26019, and the transconjugants. (B, C) S1-PFGE of these strains. Red arrows indicate transferable hybrid plasmid pCfr_tK-N. (D) PCR for blaKPC−2 and blaNDM−1 and excision sequences of CF2085, CF2085ΔNDM, CF26019, and CF26019ΔNDM. Green arrows indicate the remains of pCF2085-1 and pCF26019-1 excising of blaNDM−1-bearing surrounding in CF2085ΔNDM and CF26019ΔNDM, respectively. (E) The stability of these strains throughout the course of the 10-day continuous passage. (F) Growth curves of these strains within 25 h. [file Image_1.TIF]
